# Influence of tick age and land-use on *Borrelia burgdorferi* s.l. in *Ixodes ricinus* ticks from the Swabian Alb, Germany

**DOI:** 10.1186/s13071-025-06971-0

**Published:** 2025-09-17

**Authors:** Sara Weilage, Lidia Chitimia-Dobler, Max Müller, Martin Pfeffer, Anna Obiegala

**Affiliations:** 1 Institute of Animal Hygiene and Veterinary Public Health, University of Leipzig, An den Tierkliniken 1, 04103 Leipzig, Germany; 2 Bundeswehr Institute of Microbiology, Neuherbergstraße 11, 80937 Munich, Germany; 3 Fraunhofer Institute for Translational Medicine and Pharmacology ITMP, Immunology, Infection and Pandemic Research IIP, Munich, Germany; 4 Experimental Parasitology, Department of Veterinary Sciences, Faculty of Veterinary Medicine, Ludwig-Maximilians-Universität, LMU, Munich, Germany; 5 Institute of Evolutionary Ecology and Conservation Genomics, Ulm University, Albert-Einstein-Allee 11, 89081 Ulm, Germany; 6 Technical University of Darmstadt, Karolinenplatz 5, 64289 Darmstadt, Germany; 7 Institut d’Ecologie et des Sciences de l’Environnement Paris (IEES), Sorbonne Université, 4 Place Jussieu, 75005 Paris, France

**Keywords:** Lyme borreliosis, Tickborne pathogens, Morphometric age ratio, Biological tick age, Europe, MLST, Land-use, Biodiversity

## Abstract

**Background:**

In Europe, *Ixodes ricinus* ticks transmit various zoonotic pathogens, including *Borrelia burgdorferi* sensu lato (s.l.), the causative agent of Lyme borreliosis (LB). However, the relationship between *Borrelia* prevalence, bacterial load in unfed nymphs of different physiological ages, and the influence of season and land-use remains poorly understood. The *B. burgdorferi* s.l. complex exhibits significant genetic diversity, with genospecies varying in distribution and pathogenicity. This study aimed to examine physiological tick age in relation to land-use, *Borrelia* infection rates, and genetic diversity. Furthermore, small and large mammal diversity as well as environmental factors such as shrub cover and tree species richness were incorporated in the analyses.

**Methods:**

Ticks were collected using the flagging method on 25 experimental plots in the Biodiversity Exploratory Swabian Alb in Baden-Wuerttemberg, Germany, during spring, summer, and autumn of 2023, as well as spring 2024. This was followed by morphometric age measurement of the nymphs as well as by molecular biological analyses for *Borrelia* spp. and subsequent multilocus sequence typing (MLST) to detect *Borrelia* genospecies. Generalized linear models (GLM), and generalized linear mixed models (GLMM) were implemented to assess the effects of season and land-use on *Borrelia* prevalence and tick age and their reciprocal interactions as well as on effects of small and large mammal diversity on *Borrelia* diversity. Proportional odds logistic regression evaluated the impact of environmental factors on morphometric tick age. Model averaging was specifically applied to *Borrelia* genospecies to address uncertainty and refine coefficient estimates.

**Results:**

A total of 1,816 *Ixodes* spp. ticks were collected [63 females (3.5%), 48 males (2.6%), 1,439 nymphs (79.2%), 266 larvae (14.7%)]. The nymphs examined varied in the age groups, with age group II (young) for 1.0%, age group IV (old) accounting for 7.6% and age group III (middle-aged) for 91.4%. The overall *Borrelia* prevalence was 6.5%, but it varied among the developmental stages. The GLMM revealed that *Borrelia* prevalence in age-measured nymphs differed significantly between seasons, with the highest prevalence in autumn (11.9%; confidence intervals, CI 7.83–17.52) compared with spring (*P* = 0.0177) and summer (*P* = 0.0478). MLST analyses revealed five different genospecies: *B. garinii*, *B. afzelii*, *B. valaisiana*, *B. burgdorferi* sensu stricto and *B. lusitaniae*. For 44 samples, sequence type (ST) assignment was possible, revealing 34 different STs, all of which except for 12 have not been detected previously. Further analyses using a conditional averaged generalized linear regression model revealed a significant increase in the diversity of *Borrelia* genospecies with higher Shannon diversity indices of large mammals (*P* = 0.00824).

**Conclusions:**

Our study revealed high *Borrelia* diversity in *Ixodes ricinus* ticks in the Swabian Alb, Germany, with a peak prevalence in autumn. Large mammal diversity influenced genospecies diversity, while tree composition seemed to affect tick age, highlighting key ecological drivers of *Borrelia* transmission.

**Graphical Abstract:**

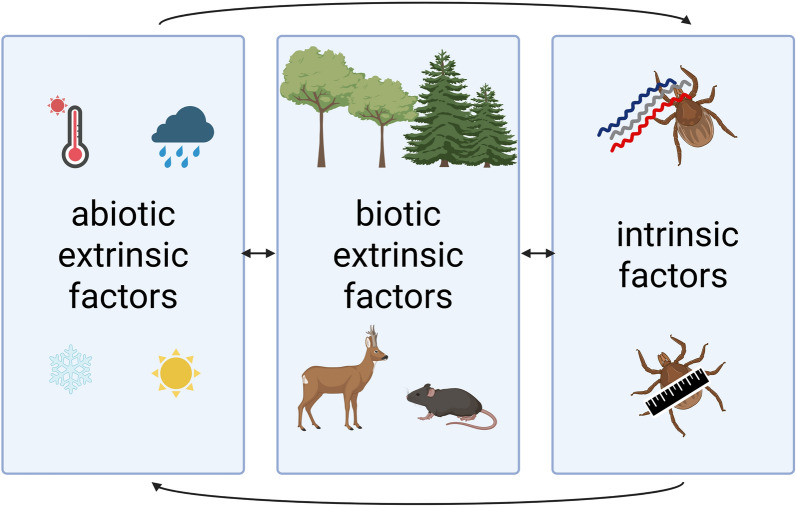

**Supplementary Information:**

The online version contains supplementary material available at 10.1186/s13071-025-06971-0.

## Background

Lyme borreliosis (LB) is the most prevalent tickborne disease in Europe, with 128,888 recorded cases annually, though estimates suggest an actual annual number of 850,000 [1, 2]. While erythema migrans is often observed in the early clinical manifestation of human LB, it can lead to severe chronic disseminated symptoms, particularly in joints, the nervous system and the heart if left untreated. The causative agents of this disease are different *Borrelia* genospecies, belonging to the *Borrelia burgdorferi* sensu lato (s.l.) complex, which comprises more than 20 genospecies, of which, a minimum of 11 occur in Europe [3]. At least seven genospecies, *B. afzelii*, *B. garinii*, *B. burgdorferi* sensu stricto, *B. bavariensis*, *B. spielmanii*, *B. valaisiana*, and *B. bissettiae* have been found to be pathogenic to humans [3], with *B. afzelii* and *B. garinii* being the most prevalent [4]. The remaining European genospecies include *B. lusitaniae*, *B. carolinensis*, *B. finlandensis*, and *B. kurtenbachii*, which are currently not considered to be pathogenic in humans. Additional genospecies occur outside Europe, such as *B. americana* and *B. andersonii* in North America, and *B. japonica* and *B. sinica* in Asia [5]. The genetic variation among different *B. burgdorferi* s.l. genospecies is largely determined by their close and often specialized associations with specific reservoir host groups, such as rodents or birds [5]. These host preferences influence which genospecies are maintained in nature and also their geographic distribution. Consequently, the distribution and habitat preferences of these reservoir hosts—shaped by environmental factors such as vegetation type, climate, and landscape structure—play a central role in determining which *Borrelia* genospecies are present within local tick populations. Thus, the occurrence of specific genospecies in vector ticks primarily reflects the ecology and availability of their reservoir hosts [6]. To persist across such a diverse range of host organisms, these Gram-negative, motile spirochetes rely on modifications in gene expression, which enable them to adapt to varying physiological environments and successfully colonize both invertebrate and vertebrate hosts [6–9]. Multilocus sequence typing (MLST) is a crucial method that categorizes *Borrelia* genospecies into sequence types (STs) to study the genetic diversity, geographic distribution, epidemiology, and population dynamics of *B. burgdorferi* s.l. [6, 10, 11]. However, detailed knowledge of habitat associations of particular STs and how they vary in tick populations on a geographical scale is still limited [6].

In Europe, not only the variety of STs in concordance to clinical manifestations is a complex scenario but also the convoluted transmission cycle of *B. burgdorferi* s.l., which includes several different host species. Ticks of the *Ixodes ricinus* complex, which belong to the family Ixodidae [12] and which are known to transmit various different zoonotic pathogens, are the main vector of *B. burgdorferi* s.l. [13, 14], with a prevalence ranging from 0% [15] up to 48.8% in Central Europe [16]. The risk of contracting *Borrelia* spp. is thus highly related to tick abundance and activity [17]. Tick activity is closely tied to physiological resources, measurable via the alloscutal/scutal index, which reflects energy reserves stored as lipids. This index—which serves as a proxy for physiological age classification—derives from body size measurements, which change in a lifespan of a tick owing to energy depletion [18–20]. Physiological age may influence *Borrelia* spp. infection probability and intensity, as these extracellular bacteria depend on host-derived nutrients [21]. In unfed ticks, nutrient deprivation over time reduces spirochaete concentration, as shown in both laboratory and field studies [20, 22]. Further, laboratory experiments indicate that *B. afzelii* infections enhance tick survival under desiccating conditions [22]. *I. ricinus* ticks are sensitive to desiccation and thrive in microclimatic environments with high humidity, making environmental factors crucial to their activity and survival. Favorable conditions are often found in mixed forests with leaf litter [23]. Thus, forest management plays a key role in influencing tick activity and potentially modulating the risk of *Borrelia* infections. Building on this understanding, this study aimed to investigate the age distribution of questing nymphal *I. ricinus* ticks across a land-use gradient in 25 forest plots in the Swabian Alb, Germany. Additionally, *Borrelia* genetic diversity was assessed in relation to tick age, season and land-use for the first time. Furthermore, factors, such as the relative abundance index (RAI) and Shannon diversity of predatory and of small and large nonpredatory mammals, were also included in the analyses.

## Methods

### Study region

The study region, Swabian Alb in Baden-Württemberg, is part of the German Biodiversity Exploratories. Spanning 420 km^2^, it features diverse landscapes and land-use types. We selected 25 of 50 forest plots covering the full range of management intensities [24]. In these forests, land-use is defined by a silvicultural management intensity index (SMI), which combines the three main components of a forest stand: stand age, stand growth, and main tree species [25] (Additional file 1: Supplementary Material Table S1) [26]. For details on the study region and plot selection, see Weilage et al. [17].

### Large mammal camera trapping

Two camera traps per plot recorded wildlife from spring 2023 to spring 2024 (9,609 camera days) following a standardized protocol [27, 28]. Images were processed with Agouti [29], and artificial intelligence-based species identification was manually verified. Human activity was excluded. A detailed description of the method can be found in Weilage et al. [17].

### Small mammal camera trapping

Custom-built small mammal camera traps [30] recorded wildlife during tick collection periods in 2023–2024. Two traps per 300-m^2^ area operated 7 days per season (984 camera days), capturing 15 s (s) videos with a 30 s delay between triggers. Species identification followed field guides [31]. For details, see Weilage et al. [17].

### Tick collection, morphometric species identification, and age measurement

Ticks were collected in spring (May), summer (August), and autumn (October) of 2023, as well as in spring (May) of 2024 using the flagging method on 300 square meters per plot. They were identified to their developmental stage and species using morphological keys [12, 32] under a stereomicroscope (Motic^®^ SMZ–171, Motic Europe, S.L.U., Barcelona, Spain). Ticks were stored in 50-ml falcons (sterile, cat. no. AN79.1, Carl Roth GmbH + Co. KG, Karlsruhe, Germany) with a blade of grass at +7 °C in the refrigerator until morphometric age measurements, which were conducted within a maximum of 1 week after collection.

The same specimens were also used in a previous study [17], which provides detailed information on the study design and sampling protocol but focused on landscape and host-related drivers of tick density and *B. burgdorferi* s.l. prevalence, while the present study investigates the influence of tick age and land-use on *B. burgdorferi* s.l.

To determine the morphometric age of ticks, body length (BL) and width (BW), as well as scutum length (SL) and width (SW) of live *I. ricinus* nymphs were first measured using an already established method [19, 20] with a Keyence VHX-900F digital microscope (Itasca, IL, USA) at 200× magnification and then incorporated into a specific formula by Uspensky et al. [19] resulting in the alloscutal/scutal index. The formula can be found in Springer et al. [20]. Previous studies have classified the values of this index into eight distinct subgroups [19], which were further assigned to three overarching categories: old (IV), middle-aged (III), and young (II) (Additional file 1: Supplementary Material Table S2) [18]. Afterwards, ticks were stored at −20 °C until further examination.

### DNA extraction of ticks and molecular analyses for *Borrelia* spp.

DNA extraction and molecular analysis via real-time polymerase chain reaction (PCR) followed established protocols and are described in detail in Weilage et al. [17, 33]. DNA was extracted using the QIAamp DNA Mini Kit (Qiagen, Hilden, Germany), and the PCR targeted the p41 flagellin gene of *Borrelia burgdorferi* sensu lato. The analytical sensitivity of the assay was ten genome copies per 10 µl, as determined by a validated standard dilution series [33]. Additionally, larvae were homogenized in 200 µl of phosphate-buffered saline (PBS), while adults were homogenized in 300 µl of PBS, as not mentioned in the original text.

To identify *Borrelia* genospecies and STs in samples with a cycle threshold (CT) value of ≤ 41, MLST was performed, targeting eight housekeeping genes *nifS*, *pyrG*, *clpX*, *pepX*, *uvrA*, *rplB*, *clpA*, and *recG*, using the GoTaq^®^ G2 Hot Start Green Master Mix (Promega GmbH, Walldorf, Germany) with slight modifications [34] to the original protocol [11, 34]. Additionally, the previously modified protocol was further adapted through several procedural adjustments, as detailed in Additional file 1: Supplementary Material Table S3. PCR products were visualized using the ultraviolet products (UVP) GelSolo Simplified ultraviolet (UV) Gel Documentation System (Analytik Jena, Germany). Sequencing was performed using the forward and reverse primers specific to each gene from the previous amplification (Eurofins Genomics, Ebersberg, Germany). Sequences were analyzed with Bionumerics software (version 7.6.1; Applied Maths, Austin, TX, USA) and compared with GenBank sequences through BLASTn (https://blast.ncbi.nlm.nih.gov/Blast.cgi). Aligned sequences were assigned to allelic ST profiles in the MLST database (http://pubmlst.org/Borrelia) with newly identified STs submitted to the curators of the pubMLST platform. For samples that did not amplify PCR products for all housekeeping genes, genospecies identification was based on at least one of the following genes: *clpA, pepX*, *recG*, and *rplB.*

### Statistical analysis

Confidence intervals (CIs; 95% CI) were calculated using the Clopper and Pearson method in GraphPad software (Graph Pad Software, SanDiego, CA). Statistical analysis was performed using R-software (version 4.1.2. for Windows; RStudio, Boston, MA) with the lme4 package [35]. A generalized linear model (GLM) with a quasibinomial error distribution and a logit link function was developed to evaluate tick age in dependence of (i) SMI (independent continuous variable), (ii) season (independent categorical variable: spring, summer, or autumn), and (iii) *Borrelia* infection status (independent binary variable; prevalence: positive: 1, negative: 0). This test was conducted for three broader *I. ricinus* nymph age groups (young, middle-aged, and old) and for eight more defined age groups according to Uspensky [19]. Furthermore, a Mann–Whitney *U* test was conducted to evaluate whether the mean morphometric age of *Borrelia*-infected ticks differs significantly from that of uninfected individuals. *Borrelia* sequence type diversity was log-transformed (log(x + 1)) to meet the assumptions of linear modeling.

In addition, a proportional odds logistic regression model (polr) was applied, using the MASS package in R, to examine the relationship between morphometric age groups (dependent variable) and variables such as SMI, canopy openness, mean tree diameter at breast height (dbh), tree species richness, shrub cover, and dead wood volume (dwv) [26, 36–39] (see Table 1). The ordinal regression model with included random effect of the plot (ordinal package using the clmm command) proved the random effect of the plot to be marginal (random effect variance: 2.398 × 10^−9^); therefore, it was not included in the final model.

**Table 1:** Dataset description: variables used for statistical analyses

Dataset	Category	Description	Unit/number	Datatype
Tick collection	Amount of ticks	Total amount of collected ticks	1,816	For details, see Weilage et al. [17]
Morphometric age and statistical analysis:	Nymphs with morphometric age estimation	Nymphal ticks with successful age determination based on Uspensky et al	1,202	
	SMI	Silvicultural management index	Index	Management [26]
	Tree species richness	Number of tree species per hectare = total tree species on the plot	Count (1/ha)	Forest structure [37–39]
	Canopy openness	Average light availability on the ground on the plot determined by terrestrial laser scanning	%	
	Mean tree dbh	Quadratic mean of the diameter at breast height of all trees with a diameter > 7 cm on the plot	cm	
	Dead wood volume	Sum of volume all lying dead wood with *Ø* > 25 cm, length > 1 m and all standing dead wood with *Ø* > 25 cm, height > 1.3 m	m^3^/ha	
	Shrub cover	Sum of each woody species cover with a height < 5 m in relation to a 20 m × 20 m area on the plot	%	
	RAI predators	Number of events per 100 camera days of species predatory species (*Martes martes*, *Meles meles*, *Mustela putorius*, *Procyon lotor*, *Vulpes vulpes)*	Index	Mammal community (mean of all seasons [17])
	H predators	Shannon’s diversity index of predatory species (*Martes martes*, *Meles meles*, *Mustela putorius*, *Procyon lotor*, *Vulpes vulpes)*	Index	
	RAI small mammals	Number of events per 100 camera days of small mammal species (*Apodemus flavicolis*, *Apodemus sylvaticus*, *Glis glis*, *Microtus arvalis*, *Muscardinus avellanarius*, *Myodes glareolus*, *Sorex araneus*, *Sorex minutus*, *Eraniceus europaeus*, *Mustela nivalis*)	Index	
	H small mammals	Shannon’s diversity index of small mammal species (*Apodemus flavicollis*, *Apodemus sylvaticus*, *Apodemus sp.*, *Glis glis*, *Microtus arvalis*, *Muscardinus avellanarius*, *Myodes glareolus*, *Sorex araneus*, *Sorex minutus*, *Erinaceus europaeus*, *Mustela nivalis)*	Index	
	RAI large mammals	Number of events per 100 camera days of large nonpredatory species (*Capreolus capreolus*, *Lepus europaeus*, *Sus scrofa*, *Sciurus vulgaris*)	Index	
	H Large mammals	Shannon’s diversity index of large nonpredatory species (*Capreolus capreolus*, *Lepus europaeus*, *Sus scrofa*, *Sciurus vulgaris*)	Index	
	S total mammals	Total species richness of all mammals	Count	
*Borrelia* analysis	Ticks in total	Total amount of ticks tested for *Borrelia burgdorferi* s.l. presence	1,651	[17]
	*Borrelia*-positive ticks	Total number of ticks classified as *Borrelia* positive after CT value threshold cut-off	108	[17]
	Ticks with identified *Borrelia* genospecies	Subset with successful genospecies determination (e.g., via sequencing)	93	
	Ticks with identified *Borrelia* sequence type (ST)	Subset with determined sequence type (e.g., MLST)	44	

This table is based on Table 1 in Weilage et al. [17]

For analyzing *B. burgdorferi* s.l. prevalence in *I. ricinus* nymphs in relation to tick age, season, and land-use (SMI), a generalized linear mixed model (GLMM) with binominal error distribution and link logit function was implemented using the lme4 package in R [35]. The model was designed to assess whether (i) tick age (independent categorical variable), (ii) season (independent nominal variable: spring, summer and autumn), and (iii) SMI (independent continuous variable) have an impact on *Borrelia* infection status (dependent binary variable; *Borrelia* abundance; positive: 1, negative: 0). The test was conducted twice, once for three broader nymph age groups (young, middle-aged, and old) and a second time for eight more defined age groups according to Uspensky [19].

Post hoc Tukey’s tests were performed to assess pairwise differences between seasons and tick age groups, adjusting for multiple comparisons. The significance threshold was set at *P* ≤ 0.05.

The *Borrelia* concentration (cp/µl) was determined by interpolating CT values against a standard curve based on known concentrations, akin to probit analysis. Unlike probit analysis, the standard dilution series was established once and later used for comparison with all positive samples, rather than being included in each PCR reaction. We used a bacterial culture of *Borrelia afzelii*, which was serially diluted in tenfold steps from an initial concentration of 2.0 × 10^5^ cells/µl down to 2.0 × 10^−1^ cells/µl, resulting in the following dilution levels: 10^5^, 10^4^, 10^3^, 10^2^, 10^1^, 10⁰, and 10^−1^.

Furthermore, a linear regression model (LM) was used to investigate how the *Borrelia* genospecies diversity is influenced by various predictors. The model was intended to evaluate whether (i) the Shannon diversity of specific host species [independent continuous variables: H_Pre (diversity of predator hosts), H_small (diversity of small mammal hosts), and H_ large (diversity of larger mammal hosts)], (ii) the relative abundance indeces (RAI) of specific host species (RAI_Pre (relative abundance index of predators), RAI_small (of small mammals), and RAI_large (of large herbivors), independent continuous variables), and (iii) total mammal host species richness (S_all, independent continuous variable) significantly affect the diversity of *Borrelia* genospecies at distinct locations.

To further explore factors influencing *Borrelia* ST diversity in ticks, another LM evaluated the impact of the same set of predictors on ST diversity.

RAIs for each species were computed as events per 100 camera days using the R script outlined in Rovero and Zimmermann [40]. The Shannon index was then derived from RAIs.

We selected and averaged the best-fitting models (ΔAICc < 2) using the MuMln package [41]. We report conditional averaged model results. All included candidate models are in the supplement.

## Results

### Descriptive overview of the dataset

A total of 1,816 *Ixodes* spp. ticks were collected, as described in Weilage et al. [17]. For the age measurement, only viable *I. ricinus* nymphs were chosen, which resulted in 1,202 out of 1,437 specimens being age classified.

In total, 1,651 ticks (*I. ricinus* and *Ixodes* spp.) (adults and nymphs from 2023 and 2024 and larvae from 2023) were tested for *B. burgdorferi* s.l. The remaining 165 ticks, comprising 162 larvae, two nymphs, and one male, were excluded from further analyses as they were used for other investigations [17]. Parameters assessed included morphometric age, *Borrelia* infection status, pathogen concentration, and genospecies diversity, as well as environmental and host-related predictors such as land-use intensity (SMI) and mammal diversity indices (Table 1).

### Nymphal age measurements

**Fig. 1:**
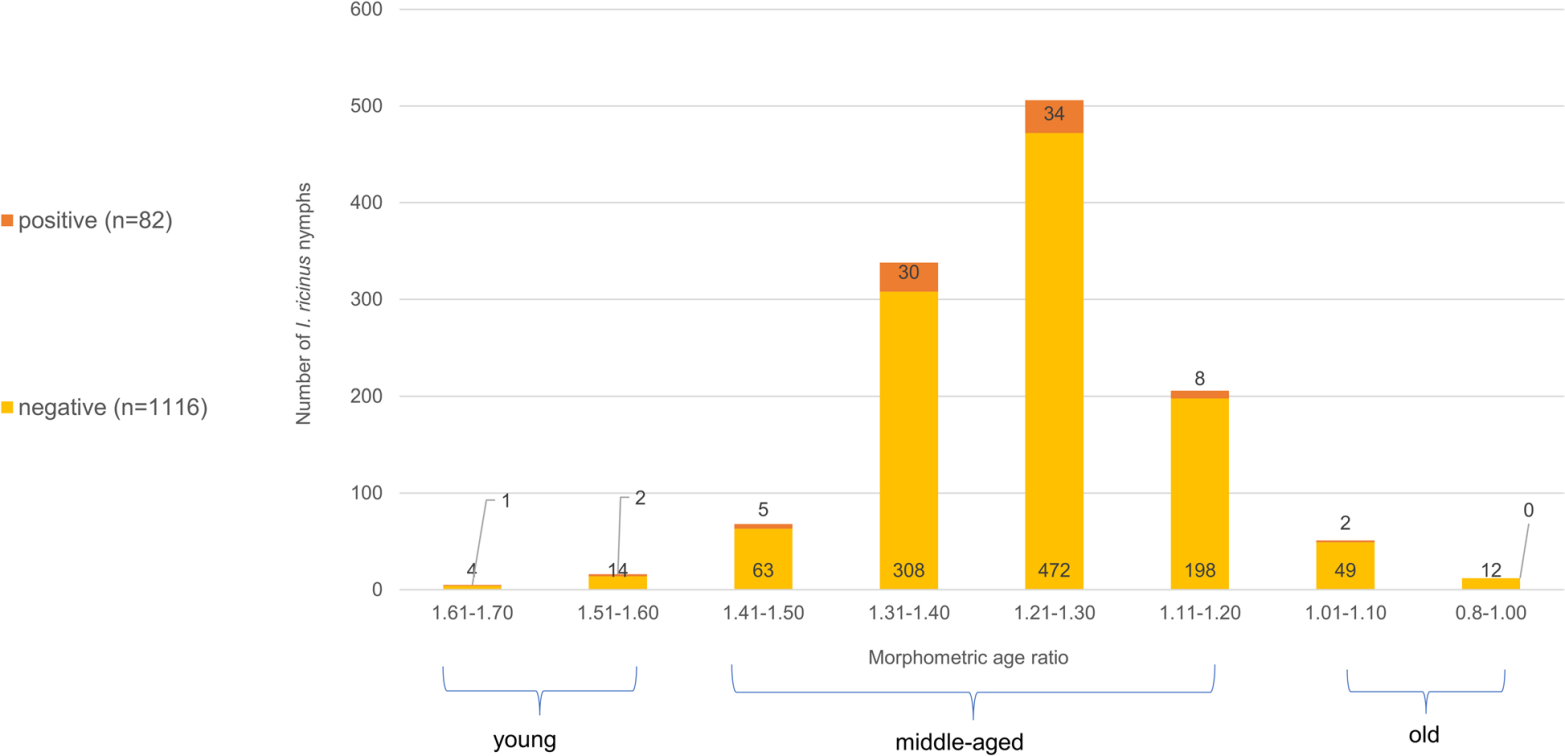
Distribution of *Borrelia burgdorferi* sensu lato (s.l.) positive and negative nymphal *Ixodes ricinus* ticks among the different age groups. Ticks were grouped according to the morphometric index: young ticks: 1.61–1.70 and 1.51–1.60; middle-aged ticks: 1.41–1.50, 1.31–1.40, 1.21–1.30 and 1.11–1.20; and old ticks: 1.01–1.10 and 0.8–1.00

**Fig. 2:**
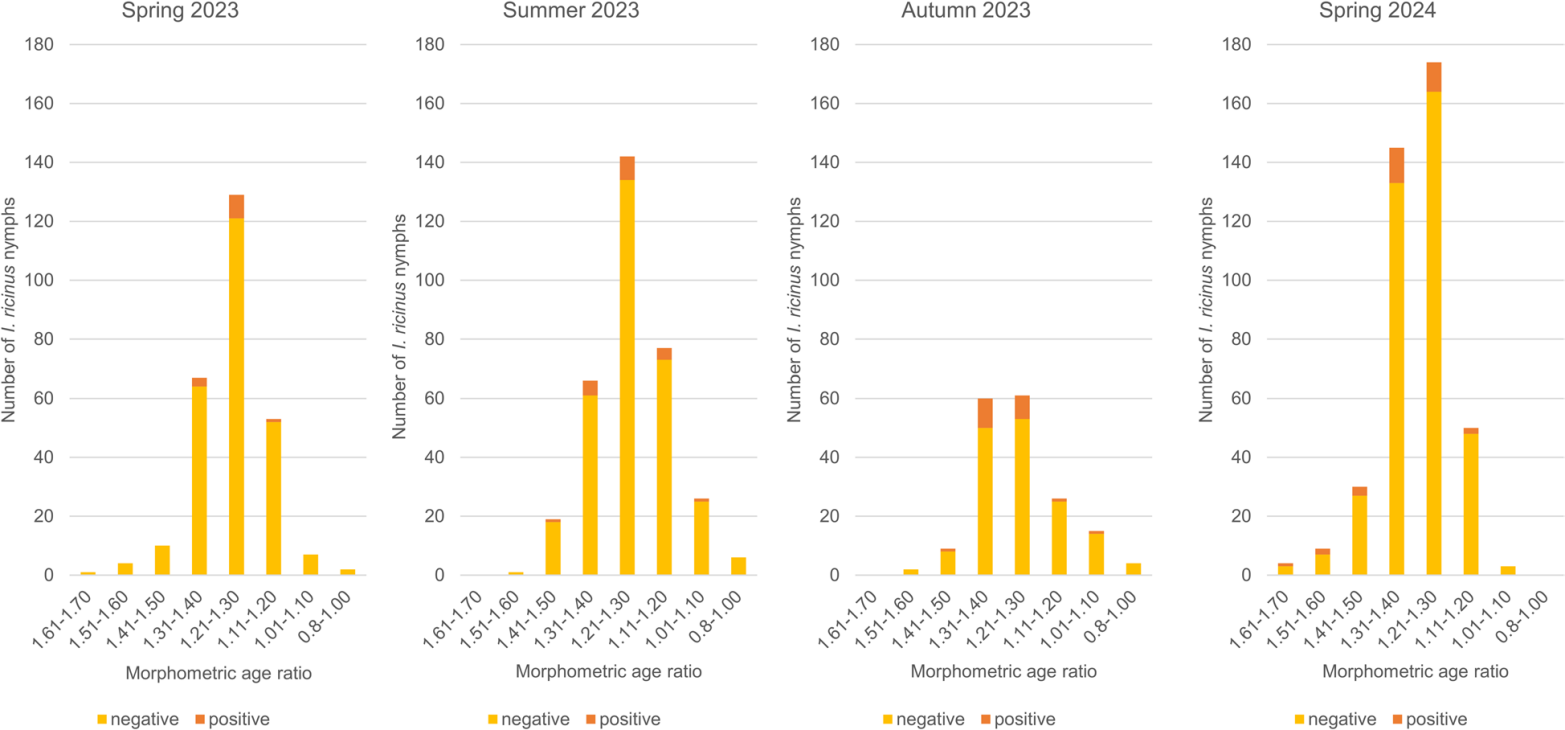
Distribution of *Borrelia burgdorferi* s.l. positive and negative nymphal *Ixodes ricinus* ticks among the different age groups and seasons (spring, summer and autumn 2023 and spring 2024). Ticks were grouped according to the morphometric index: young ticks: 1.61–1.70 and 1.51–1.60; middle-aged ticks: 1.41–1.50, 1.31–1.40, 1.21–1.30 and 1.11–1.20; and old ticks: 1.01–1.10 and 0.8–1.00

Considering the three main age groups (old, young and middle-aged), most nymphs belonged to the middle-aged category (*n* = 1118; 93.0%; CI 91.42–94.33), followed by the category of old ticks (*n* = 63; 5.2%; CI 4.1–6.7) and young ticks (*n* = 21; 1.8%; CI 1.1–2.7) (Fig. 1). A similar distribution pattern was also seen during the four individual sampling seasons (Fig. 2).

**Fig. 3:**
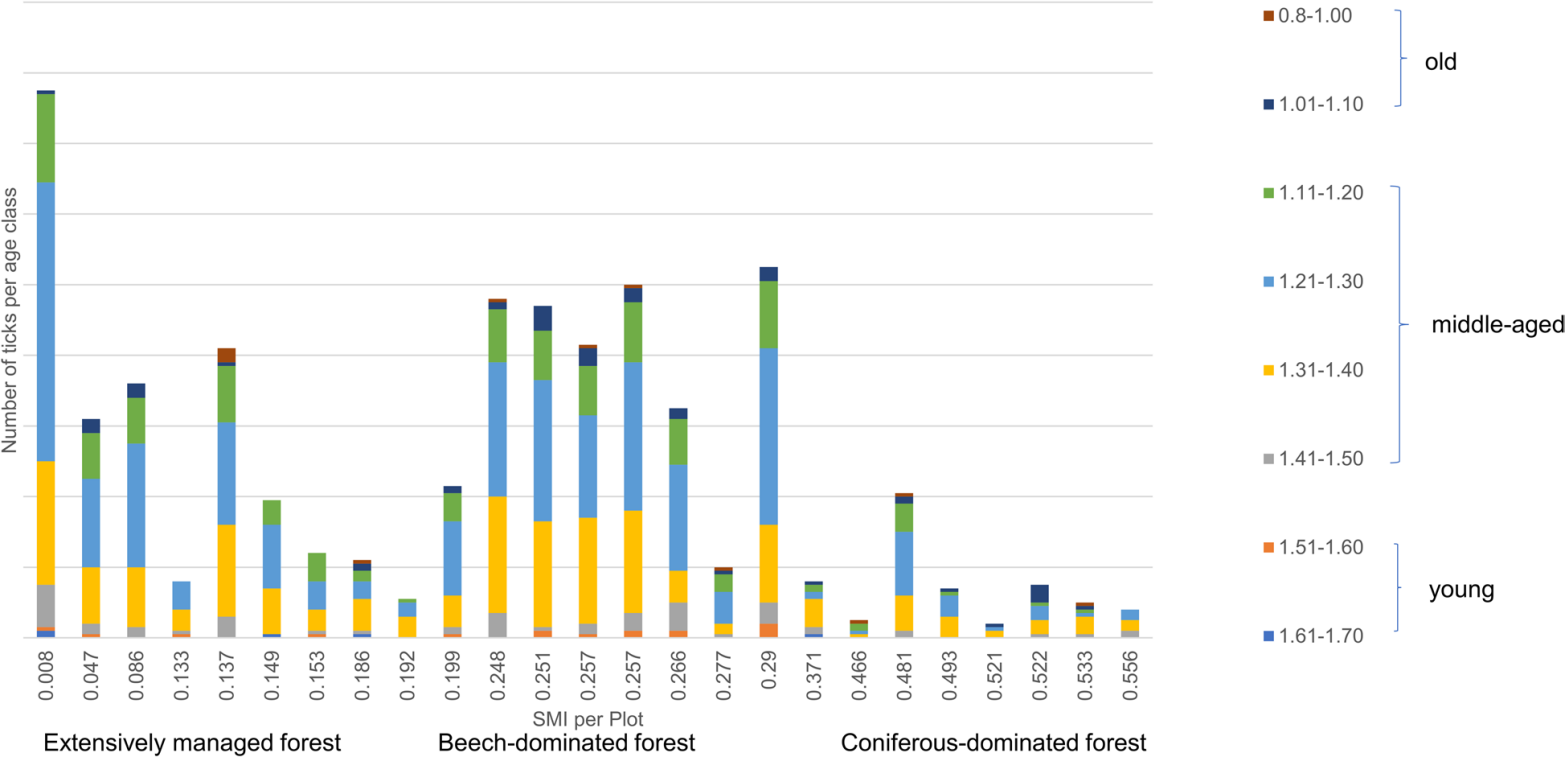
Distribution of *Ixodes ricinus* nymphs in the morphometric age groups among the different plots and their land-use intensity (silvicultural management intensity index: SMI)

A descriptive analysis revealed that the age classes exhibited similar distribution patterns across plots with varying land-use intensities (Fig. 3). Due to high residuals and a low number of individuals per age group, the GLM with eight age groups was not robust and did not allow for interpretation. The ordinal regression model revealed that none of the chosen variables (SMI, dbh, tree species richness, shrub cover, dvw, and canopy openess) exhibited significant predictions for the morphometric age groups (all *t* values < 0.32) (Additional file 1: Supplementary Material Table S4).

### Borrelia spp. prevalence and concentration

*Borrelia* DNA was detected in 112 out of 1,651 ticks (6.8%; CI 5.66–8.1), with 108 being classified as *Borrelia*-positive (6.5%; CI 5.44–7.84) after the CT value threshold cut-off [17]. The four remaining samples showed amplification in real-time polymerase chain reaction (RT-PCR) but were excluded from further analysis, as their CT values exceeded the threshold and did not allow for successful amplification of any housekeeping gene, making genospecies identification impossible. The 108 *Borrelia*-positive samples comprised nine females (14.3%; CI 7.48–25.20), seven males (14.9%; CI 7.09–28.0), and 92 nymphs (6.4%; CI 5.20–7.79) [17], of which, age determination was conducted for 82 individuals (89.1%). The remaining ten nymphs were not excluded from the *Borrelia*-related analyses but could not be included in the age-related analyses owing to missing age classification.

*Borrelia* prevalence among the eight different age groups varied between 0% (0/12; CI 0.00–28.20) and 20% (1/5; CI 2.03–64.04) (Fig. 1). The statistical analysis using GLMM with eight age groups was limited due to high residuals and thus did not allow for meaningful interpretation. However, an adjusted analysis with three age groups was successful. This revealed that, while neither tick age (age group 2: *P* = 0.17760; age group 3: *P* = 0.05388) nor land-use (SMI) (*P* = 0.64909) significantly affected the likelihood of *Borrelia* infection, season emerged as a significant factor (spring: *P* = 0.00652; summer: *P* = 0.01841) (Table 2), with the highest prevalence in autumn 2023 (11.9%; CI 7.83–17.52), followed by spring 2024 (7.2%; CI 4.52–11.29) and summer 2023 (5.6%; CI 3.59–8.69) and the lowest in spring 2023 (4.4%; CI 2.45–7.60). Comprehensive analyses have shown that ticks collected in spring exhibited a significantly lower likelihood of *Borrelia* infection compared with those collected in autumn (*P* = 0.0177), and similarly, ticks collected in summer also showed a significantly lower likelihood of infection than those collected in autumn (*P* = 0.0478). However, no significant difference in infection probability was observed between spring and summer (*P* = 0.9999) (Table 3: Post hoc of GLMM).

**Table 2:** Results of a generalized linear mixed model with binomial error distribution and logit-link function for effects of silvicultural management intensity index (SMI), season, and tick age (old, middle-aged, and young) on *Borrelia* prevalence

	Estimate	Standard error	*Z* value	Probability ( > |*z*|)
(Intercept)	−1.185995	0.710334	−1.670	0.09499
SMI	0.004166	0.009156	0.455	0.64909
Season spring	−0.787151	0.289349	−2.720	0.00652**
Season summer	−0.791710	0.335869	−2.357	0.01841*
Aged2	−0.865341	0.641865	−1.348	0.17760
Aged3	−1.870889	0.970465	−1.928	0.05388

Significance level is displayed as: P = 0 ‘***’;  0.001 ‘**’;  0.01 ‘*’; 0.05 ‘.’

**Table 3:** Results of post hoc test to compare *Borrelia* spp. infection probability between seasons

	Estimate	Standard error	*Z* value	Probability ( > |*z*|)
Spring–autumn: 0	−0.787151	0.289349	−2.720	0.0177*
Summer–autumn: 0	−0.791710	0.335869	−2.357	0.0478*
Summer–spring: 0	0.004558	0.287756	−0.016	0.9999

Significance level is displayed as: P = 0 ‘***’; 0.001 ‘**’; 0.01 ‘*’; 0.05 ‘.’

Furthermore, the GLM with three age groups indicated that *Borrelia* infection (*P* = 0.173), season (spring: *P* = 0.237; summer: *P* = 0.309, both compared with autumn), and land-use (SMI) (*P* = 0.910) had no significant influence on tick age (Table 4).

**Table 4:** Results of a generalized linear model with quasibinomial error distribution for effects of season, SMI, and *Borrelia* on tick age

	Estimate	Standard error	*Z* value	Probability ( > |*z*|)
(Intercept)	4.577274	0.860369	5.320	1.24 × 10^−7^***
Season spring	−0.916813	0.775061	−1.183	0.237
Season summer	1.278340	1.256569	1.017	0.309
SMI	0.001925	0.017080	0.113	0.910
*Borrelia*	−0.891393	0.653379	−1.364	0.173

Significance level is displayed as: P = 0 ‘***’; 0.001 ‘**’; 0.01 ‘*’; 0.05 ‘.’

Notably, the Mann–Whitney *U* test revealed no significant difference in the mean morphometric age between *Borrelia*-infected and uninfected nymphs (*U*_(*n*₁ =2,072, *n*₂ =166)_ = 165,833, *Z* = −0.8173, *P* = 0.414).

### *Borrelia* concentration

**Fig. 4:**
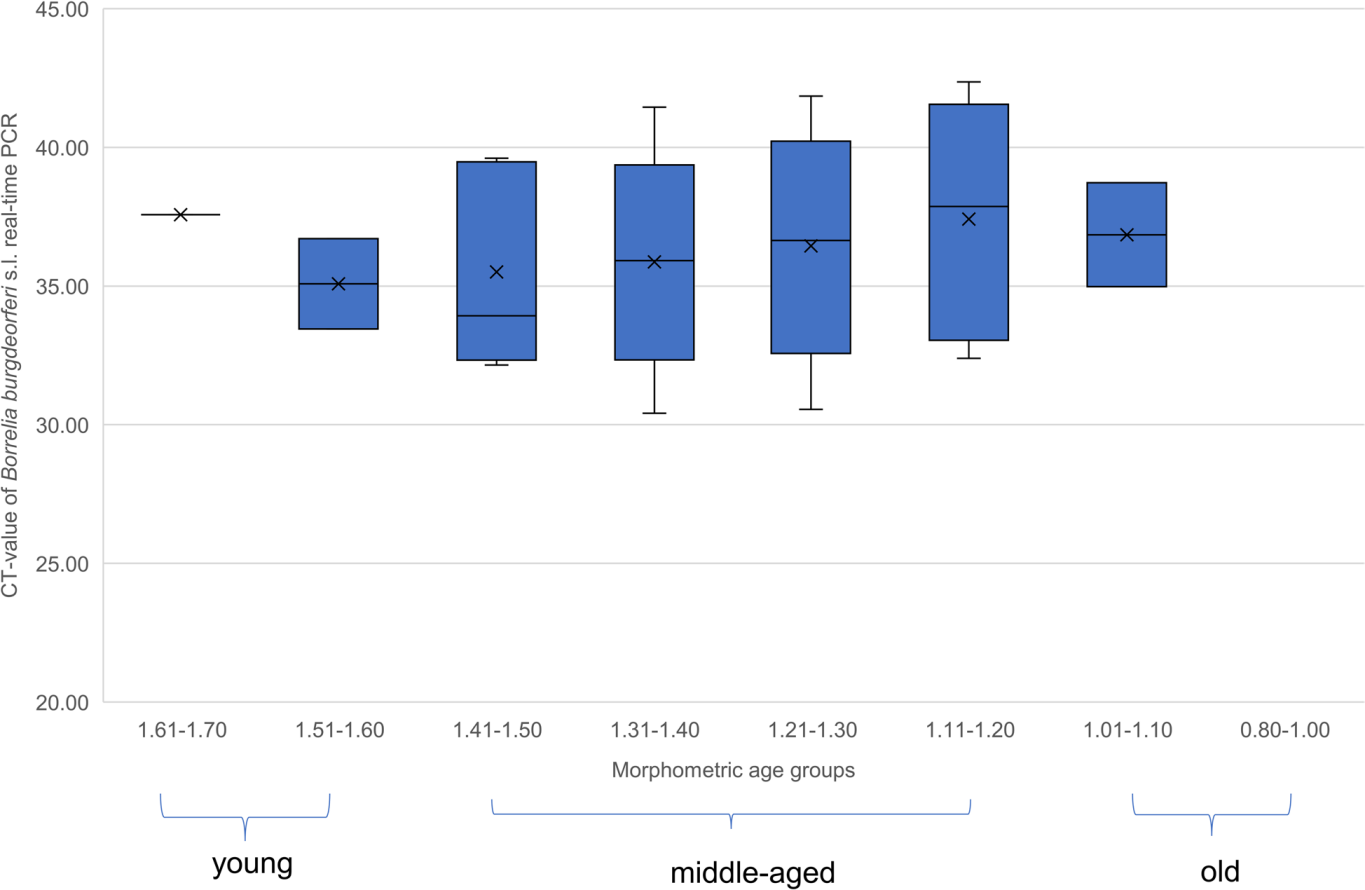
*Borrelia burgdorferi* sensu lato (s.l.) infection intensity in *Ixodes ricinus* nymphs within their different age groups. The cycle threshold (CT) is a measure of the intensity of *Borrelia* infection

The *Borrelia* concentration of the 82 positive samples from age-measured nymphs ranged between 2.00E+00 and 2.00E× 10^4^ cp/µl. In Fig. 4, the concentration is represented by the CT value of the RT-PCR, which is inversely correlated with the concentration. Accordingly, an increase in *Borrelia* concentration can be observed from the age group 1.61–1.70 (youngest) to the age group 1.41–1.50 (middle-aged), followed by a decrease in concentration in the subsequent age groups (until 1.11–1.20) (old). Finally, the concentration saturates between the last two age groups without any visible difference (1.11–1.20 and 1.01–1.10 (oldest)) (Fig. 4).

### *Borrelia* genotyping

#### Genospecies diversity

**Fig. 5:**
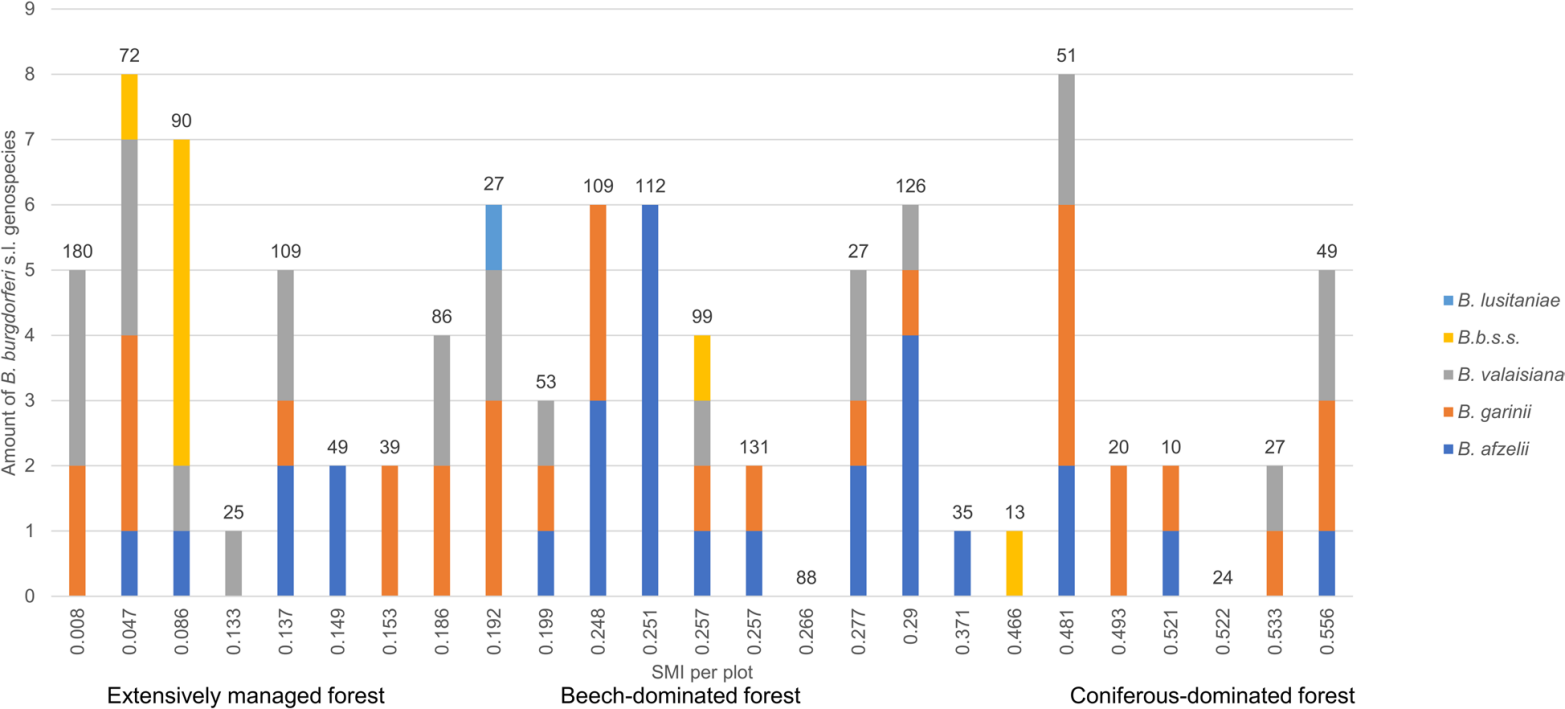
*Borrelia burgdorferi* sensu lato (s.l.) genospecies diversity among all tested plots with regard to land-use (silvicultural management intensity index: SMI). Each bar section represents the genospecies identified in an individual tick (*n* = 93). The number above each bar represents the total number of tested ticks

**Fig. 6:**
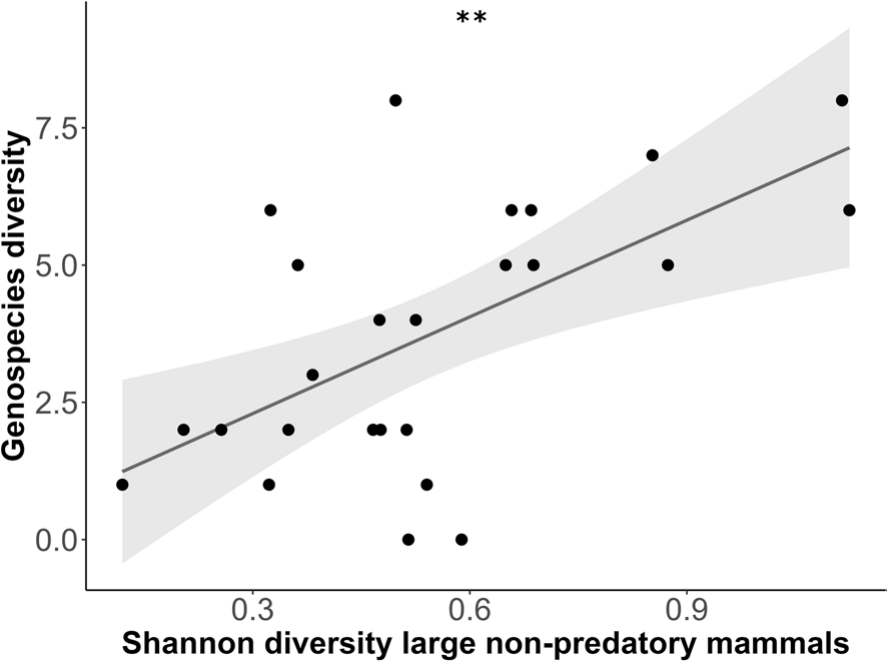
Amount of *Borrelia burgdorferi* sensu lato (s.l.) genospecies with regard to the significant factor of the conditional averaged generalized linear regression model the Shannon diversity index of large nonpredatory mammals (*P* = 0.00171**). A low Shannon diversity index represents low diversity of large nonpredatory mammals and vice versa. Each point represents the genospecies identified in an individual tick (*n* = 93)

Out of 108 samples being sequenced, full sequence typing was successfully performed for 44 samples, revealing 14 *B. valaisiana* (31.8%), 13 *B. garinii* (29.5%), 11 *B. afzelii* (25%), and six *B. burgdorferi* s.s. (13.6%). Additionally, 49 samples were assigned to genospecies via the partial sequencing of at least one gene (*clpA, pepX*, *recG*, or *rplB*) (Additional file 1: Supplementary Material Table S5), resulting in the identification of five distinct genospecies out of 93 tick samples: *B. garinii* (*n* = 31), *B. afzelii* (*n* = 29), *B. valaisiana* (*n* = 24), *B. burgdorferi* s.s. (*n* = 8), and *B. lusitaniae* (*n* = 1). The highest genospecies diversity was found at two distinct locations, each hosting four different genospecies. By contrast, the lowest diversity was observed at seven locations, where only a single genospecies was detected. No clear descriptive correlation between *Borrelia* genospecies diversity and land-use could be demonstrated (Fig. 5). However, further analysis of the influence of the mammal community through a conditional averaged generalized linear regression model revealed a significant increase in the amount of *Borrelia* genospecies with higher Shannon diversity indices of large mammals (*P* = 0.00171) (Additional file 1: Supplementary Material Tables S6–S8; Fig. 6).

#### Sequence type diversity

**Fig. 7:**
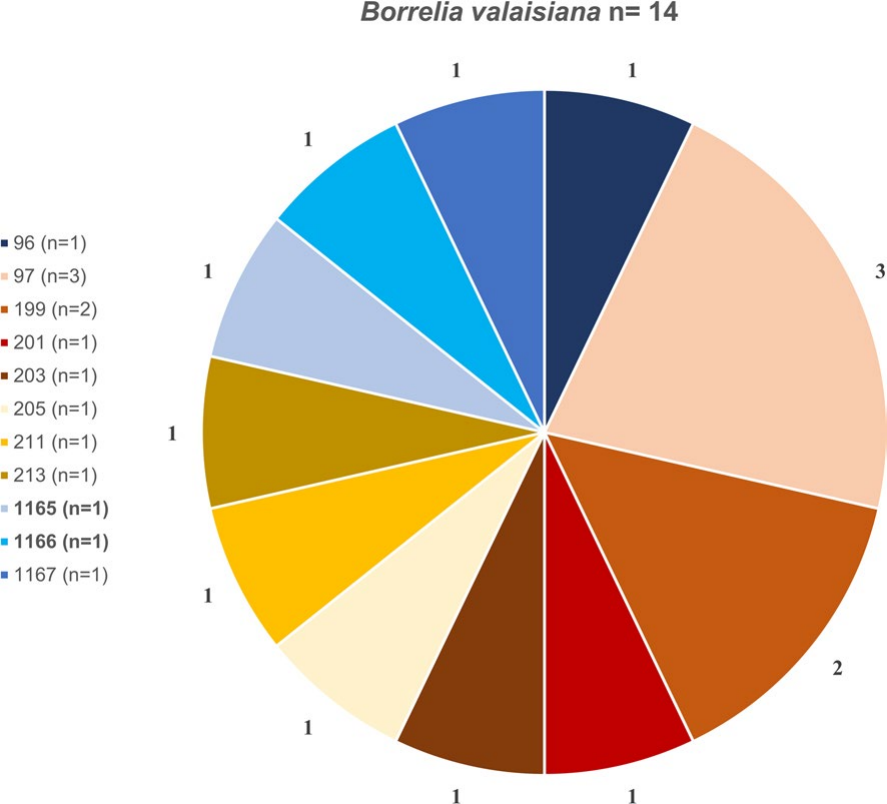
Sequence type (ST) diversity among *Borrelia valaisiana* (*n* = 14) with 11 different STs. The numbers represent the ST identifiers, *n* indicates the occurrences of each respective ST, and the percentages show the proportion of each ST among the infected ticks of the corresponding genospecies

**Fig. 8:**
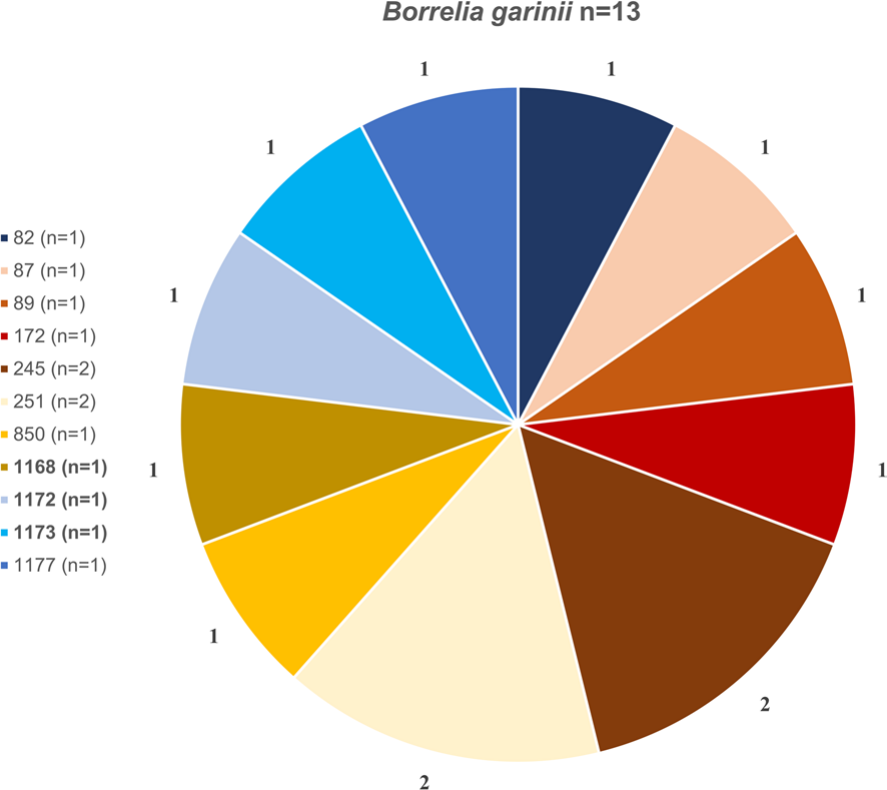
Sequence type (ST) diversity among *Borrelia garinii* (*n* = 13) with 11 different STs. The numbers represent the ST identifiers, *n *indicates the occurrences of each respective ST, and the percentages show the proportion of each ST among the infected ticks of the corresponding genospecies

**Fig. 9:**
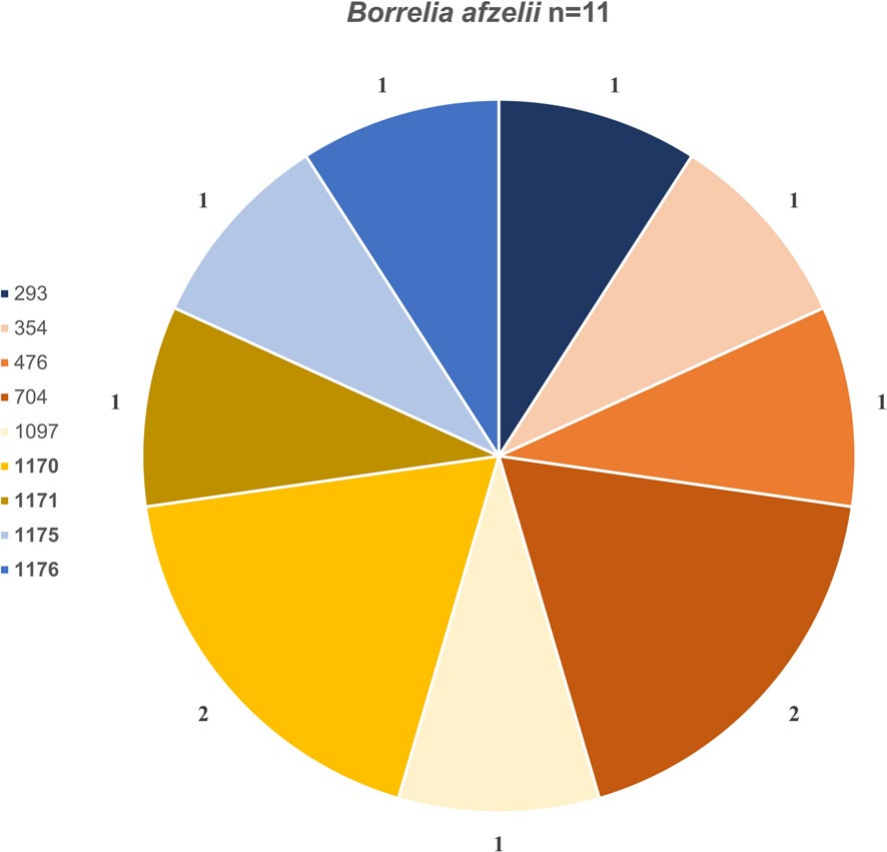
*Borrelia afzelii* (*n* = 11) with nine different STs. The numbers represent the ST identifiers, *n* indicates the occurrences of each respective ST, and the percentages show the proportion of each ST among the infected ticks of the corresponding genospecies

**Fig. 10:**
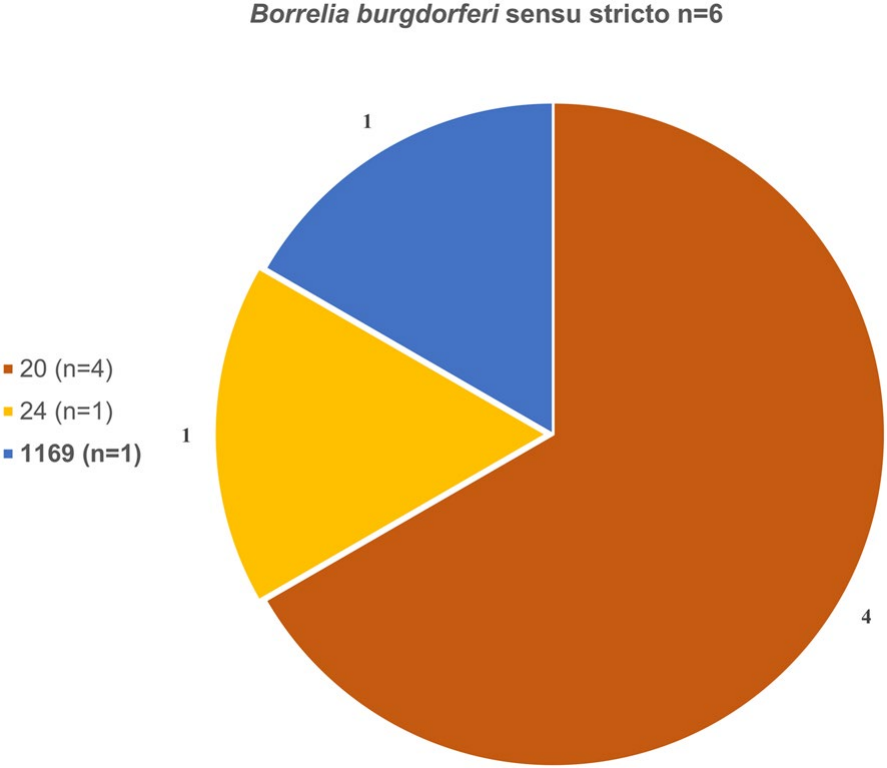
*Borrelia burgdorferi* sensu stricto (*n* = 6) with three different STs. The numbers represent the ST identifiers, *n* indicates the occurrences of each respective ST, and the percentages show the proportion of each ST among the infected ticks of the corresponding genospecies

Among the 44 fully sequenced samples, a total of 34 distinct STs were identified: 11 for *B. valaisiana*, 11 for *B. garinii*, 9 for *B. afzelii*, and 3 for *B. burgdorferi* s.s. (Figs. 7, 8, 9, 10). Notably, 12 of these STs (1165–1177) were obtained for the first time. ST 97 was the most frequently identified ST for *B. valaisiana* (*n* = 3), while ST 251 and 245 were predominant for *B. garinii* (*n* = 2), ST 704 and 1170 for *B. afzelii* (*n* = 2), and ST 20 for *B. burgdorferi* s.s. (*n* = 4) (Figs. 7, 8, 9, 10).

Although *Borrelia* genospecies were detected at 23 locations (Fig. 5), ST determination was only successful at 20 locations. The highest ST diversity was observed at three locations, each with four STs, whereas the lowest ST diversity was found at six locations, each with only one ST. A slight descriptive variation in ST diversity across different land-use types (SMI) could be observed with higher ST diversity in extensively managed forests compared with coniferous and beech forest (Table 5). A total of seven STs were detected more than once: ST 20 four times, ST 97 3 times, and STs 199, 251, 245, 704, and 1170 two times.

**Table 5:** Sequence type diversity among all tested plots with regard to land-use (silvicultural management intensity index: SMI)

Plot	SMI	Sequence types					
AEW8	0.008	205	1165				
AEW9	0.047	24	199	251	1166		
AEW7	0.086	20	20	20	20	213	1171
AEW17	0.133						
AEW50	0.137	82	211				
AEW4	0.149	476	1097				
AEW40	0.153	89	1173				
AEW39	0.186	1172					
AEW49	0.192	203					
AEW20	0.199	245					
AEW18	0.248						
AEW23	0.251	704	1176				
AEW5	0.257	199	1169				
AEW42	0.257	354					
AEW43	0.266						
AEW38	0.277	96	87	293	1167		
AEW6	0.29	97	251	1170	1170		
AEW13	0.371						
AEW11	0.466						
AEW2	0.481	201	172				
AEW3	0.493	1168					
AEW14	0.521	245	1175				
AEW1	0.522						
AEW12	0.533	97					
AEW31	0.556	97	704	850	1177		

For main tree types, see Supplementary Material Table S1

The conditional averaged generalized linear regression model investigating the impact of factors such as relative mammal abundance and diversity, and total mammal species richness on *Borrelia* ST diversity revealed no significant differences (Additional file 1: Supplementary Material Tables S9–S11).

## Discussion

*Ixodes ricinus* ticks are of great importance in the One Health sector because of their wide geographical distribution and because of their ability to transmit many different zoonotic pathogens such as *Borrelia* spp. [23, 42]. Thus far, studies that link physiological tick age with internal factors, such as *Borrelia* spp. infection status and genospecies diversity, and external factors, such as seasonality and land-use management, remain limited. This is why this study aimed to analyze tick age and the genetic diversity of *Borrelia burgdorferi* s.l. along a land-use gradient in the Swabian Alb for the first time. Regarding *I. ricinus* nymphs only, the most abundant age group found in our study was middle-aged ticks with subgroup (1.21–1.30), the fourth oldest out of eight groups, being the most represented. Overall, the abundance of ticks per age group followed a Gaussian distribution similar to findings by Springer et al. [20] who identified middle-aged ticks as the most abundant age group, with subgroup (1.21–1.30) being likewise the most representative and the oldest (0.8–1.00) and youngest (1.61–1.70) subgroups being the least prevalent overall [20]. This pattern reflects the tick’s physiological processes throughout its lifespan. Ticks store energy as lipids, which sustain essential functions such as questing during nutrient restriction [43]. As young nymphs initiating questing, they start depleting their energy reserves and thus aging [19]. This explains the lower number of young nymphs collected compared with middle-aged ones. In field conditions, the scarcity of old nymphs likely results from their cessation of host-seeking once lipid reserves drop below a critical threshold, making them undetectable in sampling [44], a phenomenon supported by two studies investigating tick lipid content, one conducted under laboratory conditions and the other in the field [44, 45]. The experimental study found significantly lower lipid levels than those observed in the field, as laboratory conditions allow for the collection of all tick age stages, regardless of their questing activity [44, 45]. However, it should be noted that the morphometric tick age is also influenced by factors such as hydration status and is thus not directly equivalent to their lipid content [44]. Taking this into account, it is equally important to examine the influence of external factors such as season and habitat on tick age [46]. Notably, one study reported accelerated ageing processes from light dry forests compared with those from dark coniferous forests [46]. However, our study found no significant effect of either land-use or season on the distribution of physiological tick age. Nevertheless, *I. ricinus* ticks are highly sensitive to desiccation [47], and avoid direct sunlight [48]. They rely on a specific microclimate with a relative humidity above 80%, which is typically found in deciduous and mixed forests with a leaf litter layer [49]. Given these ecological constraints, we considered it essential to assess how variables such as land-use (SMI), mean tree DBH, and tree species richness influence morphometric tick age. Our findings did not reveal significant effects of these factors. However, we observed a higher abundance of middle-aged ticks in extensively beech-dominated forests, while in coniferous dominated forests, tick age distribution was comparably lower. This may result from unfavorable conditions in coniferous forests [50], where the lack of leaf litter reduces humidity and microclimate stability. By contrast, the higher humidity and thicker leaf litter in extensively and beech forests likely promote tick survival [23]. To verify these patterns, further studies should investigate tick density across morphometric age groups in relation to environmental and ecological factors over multiple years, with monthly sampling.

Assessing tick age distribution alongside *B. burgdorferi* s.l. prevalence and bacterial load, no significant differences in infection prevalence between morphometric age groups was found, consistent with previous findings in *I. persulcatus* females [19] and *I. ricinus* nymphs [20]. However, prevalence was higher in older ticks, approaching significance compared with younger ones (*P* = 0.05388). Studies suggest that *B. burgdorferi* s.l.-infected *I. ricinus* nymphs have greater fat reserves [51], enhancing desiccation resistance and survival [52]. This survival advantage may explain the higher prevalence in older ticks, as prolonged host-seeking increases their likelihood of being collected by a host. *Borrelia* spp. rely entirely on host nutrients [21] and must endure nutrient scarcity in unfed nymphs. Previous studies have shown a decrease in bacterial loads from young (II) to middle-aged (III) ticks, as well as from middle-aged (III) to old (IV) ticks [19, 20]. In our case, we detected similar patterns with descriptive differences in bacterial loads of *B. burgdorferi* s.l. among the different age groups, though with an initial slight increase in bacterial loads among young ticks before the subsequent decline. To our knowledge, this is the first study to investigate *Borrelia* prevalence and diversity in ticks from the Swabian Alb, Germany. Overall, *Borrelia* DNA was detected in 6.5% of all tested ticks, with infection rates of 14.3% in females, 14.9% in males, 6.4% in nymphs, and 0% in larvae. This prevalence is notably lower than the average for Western Europe (12.3%) [53], likely due to our exclusive sampling in forest habitats, where *Borrelia* prevalence was previously found to be lower than in open landscapes such as grasslands [6]. The higher infection rates in adult ticks are probably explained by transstadial transmission and the accumulation of infection risk through multiple blood meals over their lifespan [6, 34]. Regional comparisons show substantial variation: higher adult prevalences were reported from Germany (20.4%) and Bulgaria (48.5%) [6, 54], while lower rates were found in Spain (6.1%) and Poland (10.7%) [55, 56]. Nymphal infection rates also varied, ranging from 1.4% in Spain [55] to 37% in Bulgaria [54], with our value (6.4%) aligning closely with data from Poland (6.3%) [56] and below those from Germany (11.1%), [6] and France (12.2%) [47]. In our study, the detected overall *B. burgdorferi* s.l. prevalence of 6.8% in measured nymphal *I. ricinus* ticks showed significant seasonal variation. The highest prevalence was recorded in autumn, while notably lower prevalences were observed in spring and summer. Previous studies have shown contrasting findings. One of them recorded no seasonal variations [57] during a period of 15 years [57]. Other studies found higher estimated prevalences in summer compared with spring [58] and also autumn [58, 59]. Seasonal host availability and infection rate fluctuations may drive *Borrelia* seasonality. Since *B. burgdorferi* s.l., including *B. afzelii*, *B. garinii*, and *B. burgdorferi* s.s. are rarely transmitted transovarial [60, 61], the pathogen cycle is mostly interrupted at the egg stage, making the infection dynamics dependent on larvae feeding on infectious hosts. The higher *Borrelia* prevalence observed in autumn likely reflects complex seasonal dynamics. Favorable spring and summer conditions promote increased activity of both ticks and hosts, leading to more host–tick interactions. Although the rise in small mammal reproduction during summer does not immediately increase the number of infectious hosts—since juveniles must first acquire infection—overall host density and the presence of persistently infected, longer-lived individuals may raise the chances of larvae feeding on infectious hosts. These infected larvae then molt and become nymphs, which begin questing later in the season, potentially explaining the accumulation of *Borrelia*-infected ticks by autumn. A study from the Netherlands reported a steady increase in *Borrelia* prevalence among nymphs from March to August, which is similar to our findings. Seasonal fluctuations in host availability and infection rates were discussed as possible drivers of this pattern [59]. However, regional variation suggests that local host communities, climate, and habitat conditions modulate these dynamics, warranting further comparative research.

Our study identified five different *Borrelia* genospecies being present in the Swabian Alb, with *B. garinii* being the most prevalent followed by *B. afzelii, B. valaisiana*, *B. burgdorferi* s.s., and a *B. lusitaniae*. These results are in line with findings from a further meta-analysis, which identified *B. afzelii*, *B. garinii*, and *B. valaisiana* being the most prevalent genospecies in Europe [62]. Other German studies from 2020 and 2022 have also shown higher prevalence rates of *B. afzelii* and *B. garinii* and low prevalence rates of *B. lusitaniae* and *B. burgdorferi* s.s. in *I. ricinus* ticks [6, 20, 63]. Different *Borrelia* genospecies are known to be associated with specific reservoir hosts. *Borrelia afzelii* is primarily linked to rodents, while *B. garinii* and *B. valaisiana* are associated with ground-feeding birds. Rodents and birds, which serve as primary reservoir hosts for *B. burgdorferi* s. s. [5], are commonly found across various habitats, including European forests [64]. Boulanger et al. [47] examined the impact of land-use, soil type, and forest stand type on *I. ricinus* and its associated pathogens, revealing that the distribution of *Borrelia* genospecies is shaped by their specific host associations, which in turn are influenced by habitat characteristics [47]. In their study, *Borrelia afzelii* was predominantly found in dense forests, *B. garinii* occurred more often in open landscapes, *B. burgdorferi* s.s. appeared less specifically, and *B. spielmanii* thrived in mixed forests [47]. Further research found *B. garinii* and *B. burgdorferi* s.s. more common in oak forests, whereas *B. afzelii* prevailed in pine stands [65], emphasizing the role of forest composition in *Borrelia* genospecies distribution. We observed distinct patterns in the distribution of *Borrelia* genospecies across different forest types. *B. valaisiana* and *B. burgdorferi* s.s. were most frequently found in extensively managed forests, *B. afzelii* in beech-dominated forests, and *B. garinii* in coniferous forests. The high occurrence of *B. afzelii* in beech forests may be explained by the fact that rodents find an important food source in beech fruits, leading to population peaks, particularly after mast years [66]. By contrast, birds have a larger range of movement and are not necessarily dependent on local food availability, but through their ground contact while foraging, they have a high likelihood of coming into contact with ticks and thus likely becoming infected with *Borrelia* [67]. This could explain why bird-associated *Borrelia* genospecies, such as *B. garinii* and *B. valaisiana*, can be found more frequently in other habitats. These different associations of the genospecies could also explain the ambiguous effects of the small mammal community on *Borrelia* prevalence in Weilage et al. [17], where all genospecies were summarized in the analysis. However, further analyses in our study revealed that large mammal diversity significantly influences the number of genospecies, with greater diversity in large mammals correlating with an increase in genospecies diversity. Large mammals, particularly ungulates such as deer, play a crucial role in sustaining tick populations by serving as primary blood hosts for adult *I. ricinus* ticks [68], thereby maintaining high tick densities and facilitating the circulation of multiple *Borrelia* genospecies. These findings suggest that host community composition and ecosystem structure, rather than mere host abundance, are critical factors influencing the diversity of *Borrelia* genospecies. It should be noted, however, that the explanatory power of this approach is inherently limited, as it considers only mammalian host species richness. This is a noteworthy limitation, given that several *B. burgdorferi* sensu lato genospecies are primarily associated with nonmammalian hosts, such as birds or reptiles.

Our study revealed 34 different STs, including 12 STs that were never detected before. Notably, the ST diversity in extensively managed forests seems to be higher compared with beech- and coniferous-dominated forests. This observation can likely be attributed to the higher biodiversity at these sites. All of the known STs were previously found at least in ticks, several of them also in humans. STs of individual *Borrelia* genospecies could arise through recombination, potentially allowing the organism to adapt to external and internal conditions [69]. Therefore, we assume that transmission mechanisms and spatial distribution are highly complex. The occurrence of many different STs in our study reflects a very heterogeneous evolutionary dynamic, indicating rapid adaptation and high genetic flexibility among the Swabian Alb. However, this was not attributable to the mammalian host community in our study.

## Conclusions

Our study demonstrates high *Borrelia* genospecies diversity in *I. ricinus* ticks among distinct locations in the Swabian Alb, Germany. We showed a seasonal fluctuation of *Borrelia* prevalence in *I. ricinus* nymphs with higher prevalences in autumn compared with spring and summer. Moreover, greater large mammal diversity correlated with higher *Borrelia* genospecies diversity. While tick age structure remained consistent along the land-use gradient, it appeared to be influenced by tree species composition. With all these results stated, we must also point out the limits of the study, as it must be noted that these results were only observed within 1 year. This is why it would be necessary in the future to observe these fluctuations long-term. Further, it must be noted that the mammal diversity as well as the tick age determination can only be regarded as a proxy, as the methods used to describe these factors were not fully comprehensive. Nonetheless, the results of the study underscore the complexity of the life cycle of *Borrelia* spp. and the need for further research on the ecological drivers of *Borrelia* transmission.

## Supplementary Information

**Additional file 1: Table S1.** Silvicultural management intensity index (SMI) per plot. **Table S2.** Age groups according to Balashov et al. and Uspensky et al. [18]. **Table S3.** Modified amplification steps with regard to the original modified protocol **Table S4.** Results of the ordinal regression model for effects of SMI, canopy openness, mean tree dbh (diameter at breast height), tree species richness, shrub and dwv (dead wood volume) on morphometric tick age. **Table S5.** Sequencing results based on the clpA, pepX, recG, or rplB genes in *Ixodes ricinus* samples that did not yield a complete multilocus sequence typing (MLST) profile. **Table S6.** Results of the full linear regression model for effects of the relative abundance indices and Shannon diversities of predators, small and large mammals as well as the total species richness of mammals on *Borrelia *genospecies diversity. Significance: 0 ‘***’ 0.001 ‘**’ 0.01 ‘*’ 0.05 ‘.’ 0.1 ‘ ’ 1. **Table S7** Conditional averaged linear regression results using a normal distribution for effects of Shannon diversity of larger non-predatory mammals and predators and total species richness of mammals on *Borrelia *genospecies diversity. Significant variables are marked with an*. **Table S8.** AICc table for the candidate models describing *Borrelia* genospecies diversity, displaying the models with delta AICc<2 (Full model: *Borrelia* genospecies diversity ~ RAI_Pre + H_Pre + RAI_large + H_large + RAI_small + H_small + S_all). **Table S9.** Results of the full linear regression model for effects of the relative abundance indices and Shannon diversities of predators, small and large mammals as well as the total species richness of mammals on *Borrelia* sequence type diversity. **Table S10.** Conditional averaged linear regression results using a normal distribution for effects of Shannon diversity of larger non-predatory mammals on *Borrelia* sequence type diversity. **Table S11***. *AICc table for the candidate models describing *Borrelia* sequence type (ST) diversity, displaying the models with delta AICc<2 (Full model: *Borrelia *ST diversity ~ RAI_Pre + H_Pre + RAI_large + H_large + RAI_small + H_small + S_all)

## Data Availability

Tick collection and mammal trapping: data on tick collection and small and large mammal trapping were made available from Weilage et al. [17]. This work is based on data elaborated by the Biodiversity Exploratories program (DFG Priority Program 1374). The datasets are publicly available in the Biodiversity Exploratories Information System (10.17616/R32P9Q). The datasets are listed in the references section. However, to give data owners and collectors time to perform their analysis, the Biodiversity Exploratories’ data and publication policy includes by default an embargo period of 3 years from the end of data collection/data assembly, which applies to the remaining datasets (IDs: 32109 [70], 32111 [71], 32113 [72]). These datasets will be made publicly available via the same data repository. Landscape data: [36]. Silvicultural management index (SMI): [26]. Local forest structure: [37]. Shrub cover: [38]. Canopy openness: [39].

## Abbreviations

LB: Lyme borreliosis
B: *Borrelia*
s.l.: Sensu lato
MLST: Multilocus sequence typing
GLMM: Generalized linear mixed model
GLM: Generalized linear model
LM: Linear model
SMI: Silvicultural management intensity index
AI: Artificial intelligence
S: Seconds
km^2^: Square kilometers
m^2^: Square meters
BL: Body length
BW: Body width
SL: Scutum length
SW: Scutum width
DNA: Deoxyribonucleic acid
PCR: Polymerase chain reaction
PBS: Phosphate-buffered saline
CT: Cycle threshold
UVP: Ultraviolet products
UV: Ultraviolet
BLASTn: Basic local alignment search tool for nucleotides
CI: Confidence interval
Polr: Proportional odds logistic regression model
DBH: Diameter at breast height
Dwv: Dead wood volume
Cp/µl: Copy-numbers per microliter
H_pre: Diversity of predator hosts
H_small: Diversity of small mammal hosts
H_large: Diversity of larger mammal hosts
RAI: Relative abundance index
S_all: Total mammal host species richness
ΔAICc: Delta Akaike information criterion
RT-PCR: Real-time polymerase chain reaction
