# Supplementary material for: Influence of tick age and land-use on Borrelia burgdorferi s.l. in Ixodes ricinus ticks from the Swabian Alb, Germany
Source: Parasit Vectors. 2025 Sep 17;18:370. doi: 10.1186/s13071-025-06971-0 (PMC12445037; doi:10.1186/s13071-025-06971-0)
Supplement: Supplementary file 1 — Additional file 1: Table S1. Silvicultural management intensity index (SMI) per plot. Table S2. Age groups according to Balashov et al. and Uspensky et al. [18]. Table S3. Modified amplification steps with regard to the original modified protocol Table S4. Results of the ordinal regression model for effects of SMI, canopy openness, mean tree dbh (diameter at breast height), tree species richness, shrub and dwv (dead wood volume) on morphometric tick age. Table S5. Sequencing results based on the clpA, pepX, recG, or rplB genes in Ixodes ricinus samples that did not yield a complete multilocus sequence typing (MLST) profile. Table S6. Results of the full linear regression model for effects of the relative abundance indices and Shannon diversities of predators, small and large mammals as well as the total species richness of mammals on Borrelia genospecies diversity. Significance: 0 ‘***’ 0.001 ‘**’ 0.01 ‘*’ 0.05 ‘.’ 0.1 ‘ ’ 1. Table S7 Conditional averaged linear regression results using a normal distribution for effects of Shannon diversity of larger non-predatory mammals and predators and total species richness of mammals on Borrelia genospecies diversity. Significant variables are marked with an*. Table S8. AICc table for the candidate models describing Borrelia genospecies diversity, displaying the models with delta AICc<2 (Full model: Borrelia genospecies diversity ~ RAI_Pre + H_Pre + RAI_large + H_large + RAI_small + H_small + S_all). Table S9. Results of the full linear regression model for effects of the relative abundance indices and Shannon diversities of predators, small and large mammals as well as the total species richness of mammals on Borrelia sequence type diversity. Table S10. Conditional averaged linear regression results using a normal distribution for effects of Shannon diversity of larger non-predatory mammals on Borrelia sequence type diversity. Table S11. AICc table for the candidate models describing Borrelia sequence type (ST) diversity, dis [file 13071_2025_6971_MOESM1_ESM.docx]

**Supplementary information: Additional file 1**

**Table S1.** Silvicultural management intensity index (SMI) per plot

| **Plot** | **SMI** | **Main tree species** | |
| --- | --- | --- | --- |
| AEW1 | 0.52 | Coniferous forest |  |
| AEW2 | 0.48 | Coniferous forest |  |
| AEW3 | 0.49 | Coniferous forest |  |
| AEW4 | 0.15 | Beech forest |  |
| AEW5 | 0.26 | Beech forest |  |
| AEW6 | 0.29 | Beech forest |  |
| AEW7 | 0.09 | Beech forest |  |
| AEW8 | 0.01 | Beech forest |  |
| AEW9 | 0.05 | Beech forest |  |
| AEW11 | 0.47 | Coniferous forest |  |
| AEW12 | 0.53 | Coniferous forest |  |
| AEW13 | 0.37 | Coniferous forest |  |
| AEW14 | 0.52 | Coniferous forest |  |
| AEW17 | 0.13 | Beech forest |  |
| AEW18 | 0.25 | Beech forest |  |
| AEW20 | 0.20 | Beech forest |  |
| AEW23 | 0.25 | Beech forest |  |
| AEW31 | 0.56 | Coniferous forest |  |
| AEW38 | 0.27 | Beech forest |  |
| AEW39 | 0.19 | Beech forest |  |
| AEW40 | 0.15 | Beech forest |  |
| AEW42 | 0.26 | Beech forest |  |
| AEW43 | 0.28 | Beech forest |  |
| AEW49 | 0.19 | Beech forest |  |
| AEW50 | 0.14 | Beech forest |  |

**Table S2** Age groups according to Balashov et al. [18] and Uspensky et al. [19]

| Age Group according to Balashov [1] | Group IV (old) | Group III  (middle-aged) | Group II  (young) |
| --- | --- | --- | --- |
| Alloscutal/ | 0.8-1.00 | 1.11-1.20 | 1.51-1.60 |
| Scutal | 1.01-1.10 | 1.21-1.30 | 1.61-1.70 |
| Index [2] |  | 1.31-1.40 |  |
|  |  | 1.41-1.50 |  |

**Table S3.** Modified amplification steps with regard to the original modified protocol [34].

| **Gene** | **1. amplification** | | | | **2. amplification** | | | |
| --- | --- | --- | --- | --- | --- | --- | --- | --- |
|  | Annealing 1 | | Annealing 2 | | Annealing 1 | | Annealing 2 | |
|  | Tempera-ture (°C) | Cycles | Tempera-ture (°C) | Cycles | Tempera-ture (°C) | Cycles | Tempera-ture (°C) | Cycles |
| *nifS* | 51-43 touch-down | 9 | 46 | 30 | 51 | 35 |  |  |
| *pyrG* | 47 | 39 |  |  | 49 | 35 |  |  |
| *clpX* | 51-43 touch-down | 9 | 46 | 30 | 51-43 touch-down | 9 | 46 | 30 |
| *pepX* | 51-43 touch-down | 9 | 46 | 30 | 55 | 35 |  |  |
| *uvrA* | 51 | 39 |  |  | 51 | 35 |  |  |
| *rplB* | 51-43 touch-down | 9 | 46 | 30 | 51-43 touch-down | 9 | 46 | 30 |
| *clpA* | 47 | 39 |  |  | 51-43 touch-down | 9 | 46 | 30 |
| *recG* | 55 | 39 |  |  | 50 | 35 |  |  |

**Table S4**. Results of the ordinal regression model for effects of SMI, canopy openness, mean tree dbh (diameter at breast height), tree species richness, shrub and dwv (dead wood volume) on morphometric tick age.

|  | Value | Std. Error | t value |
| --- | --- | --- | --- |
| SMI | 0.284532 | 0.889032 | 0.3200 |
| Canopy openness | -0.006166 | 0.024462 | -0.2521 |
| Mean tree dbh | 0.001785 | 0.009794 | 0.1823 |
| Species richness | -0.006069 | 0.034687 | -0.1750 |
| Shrub | -0.001120 | 0.005315 | -0.2107 |
| DWV | 0.000957 | 0.003489 | 0.2741 |

**Table S5.** Sequencing results based on the clpA, pepX, recG, or rplB genes in *Ixodes ricinus* samples that did not yield a complete multilocus sequence typing (MLST) profile.

| Sample | | | Identi-fied  gene | *Borrelia*  genospecies | | Most similar  sequence | |
| --- | --- | --- | --- | --- | --- | --- | --- |
| Develop-mental stage | Host species | Collection site |  |  | Identity (%) | | GenBank ID |
| Nymph | *I. ricinus* | AEW6 | recG | *B. afzelii* | 100 | | MG972813 |
| Nymph | *I. ricinus* | AEW13 | recG | *B. afzelii* | 100 | | JX971362 |
| Nymph | *I. ricinus* | AEW23 | clpA | *B. afzelii* | 100 | | CP075442 |
| Nymph | *I. ricinus* | AEW2 | rplB | *B. afzelii* | 100 | | CP075249 |
| Nymph | *I. ricinus* | AEW5 | recG | *B. afzelii* | 100 | | CP075448 |
| Nymph | *I. ricinus* | AEW18 | rplB | *B. afzelii* | 100 | | CP075249 |
| Nymph | *I. ricinus* | AEW18 | recG | *B. afzelii* | 100 | | CP075249 |
| Nymph | *I. ricinus* | AEW20 | recG | *B. afzelii* | 100 | | MG972813 |
| Nymph | *I. ricinus* | AEW23 | rplB | *B. afzelii* | 100 | | CP075249 |
| Nymph | *I. ricinus* | AEW23 | recG | *B. afzelii* | 100 | | MG972813 |
| Nymph | *I. ricinus* | AEW23 | rplB | *B. afzelii* | 100 | | CP075249 |
| Nymph | *I. ricinus* | AEW38 | recG | *B. afzelii* | 100 | | MG972813 |
| Nymph | *I. ricinus* | AEW50 | pepX | *B. afzelii* | 100 | | MH747528 |
| Nymph | *I. ricinus* | AEW6 | recG | *B. afzelii* | 99.85 | | CP075249 |
| Nymph | *I. ricinus* | AEW9 | recG | *B. afzelii* | 99.85 | | CP075249 |
| Nymph | *I. ricinus* | AEW18 | pepX | *B. afzelii* | 99.82 | | CP075249 |
| Nymph | *I. ricinus* | AEW2 | recG | *B. afzelii* | 99.54 | | JX971362 |
| Nymph | *I. ricinus* | AEW50 | pepX | *B. afzelii* | 99.47 | | CP075249 |
| Male | *I. ricinus* | AEW11 | pepX | *B. burgdorferi* sensu stricto | 100 | | CP094597 |
| Nymph | *I. ricinus* | AEW7 | recG | *B. burgdorferi* sensu stricto | 100 | | CP094597 |
| Nymph | *I. ricinus* | AEW2 | clpA | *B. garinii* | 100 | | CP075218 |
| Nymph | *I. ricinus* | AEW8 | recG | *B. garinii* | 100 | | CP075222 |
| Nymph | *I. ricinus* | AEW12 | pepX | *B. garinii* | 100 | | CP075222 |
| Nymph | *I. ricinus* | AEW49 | clpA | *B. garinii* | 100 | | CP075424 |
| Nymph | *I. ricinus* | AEW3 | recG | *B. garinii* | 100 | | CP075222 |
| Nymph | *I. ricinus* | AEW49 | pepX | *B. garinii* | 100 | | CP075218 |
| Male | *I. ricinus* | AEW5 | recG | *B. garinii* | 100 | | CP075219 |
| Nymph | *I. ricinus* | AEW18 | recG | *B. garinii* | 100 | | CP075220 |
| Nymph | *I. ricinus* | AEW18 | recG | *B. garinii* | 100 | | CP075220 |
| Nymph | *I. ricinus* | AEW18 | clpA | *B. garinii* | 100 | | CP075218 |
| Nymph | *I. ricinus* | AEW39 | pepX | *B. garinii* | 100 | | AB555937 |
| Male | *I. ricinus* | AEW2 | rplB | *B. garinii* | 99.84 | | AB555988 |
| Nymph | *I. ricinus* | AEW8 | recG | *B. garinii* | 99.69 | | CP075219 |
| Female | *I. ricinus* | AEW2 | recG | *B. garinii* | 99.58 | | CP075217 |
| Nymph | *I. ricinus* | AEW9 | recG | *B. garinii* | 99.54 | | CP075420 |
| Nymph | *I. ricinus* | AEW42 | recG | *B. garinii* | 99.54 | | CP075420 |
| Nymph | *I. ricinus* | AEW49 | recG | *B. garinii* | 99.54 | | CP075420 |
| Nymph | *I. ricinus* | AEW9 | pyrG | *B. garinii* | 87.96 | | CP075219 |
| Nymph | *I. ricinus* | AEW49 | recG | *B. lusitaniae* | 100 | | MH747538 |
| Nymph | *I. ricinus* | AEW8 | recG | *B. valaisiana* | 100 | | CP009117 |
| Male | *I. ricinus* | AEW31 | rplB | *B. valaisiana* | 99.84 | | CP009117 |
| Nymph | *I. ricinus* | AEW17 | rplB | *B. valaisiana* | 99.83 | | CP009117 |
| Nymph | *I. ricinus* | AEW39 | recG | *B. valaisiana* | 99.69 | | CP009117 |
| Nymph | *I. ricinus* | AEW49 | recG | *B. valaisiana* | 99.69 | | CP009117 |
| Nymph | *I. ricinus* | AEW50 | pepX | *B. valaisiana* | 99.69 | | CP009117 |
| Nymph | *I. ricinus* | AEW39 | pepX | *B. valaisiana* | 99.66 | | CP009117 |
| Female | *I. ricinus* | AEW20 | recG | *B. valaisiana* | 99.58 | | CP009117 |
| Nymph | *I. ricinus* | AEW2 | pepX | *B. valaisiana* | 99.37 | | CP009117 |
| Nymph | *I. ricinus* | AEW9 | recG | *B. valaisiana* | 99.19 | | CP009117 |

**Table S 6.** Results of the full linear regression model for effects of the relative abundance indices and Shannon diversities of predators, small and large mammals as well as the total species richness of mammals on *Borrelia* genospecies diversity.

|  | Estimate | Std. Error | t value | Probability (>\|t\|) |
| --- | --- | --- | --- | --- |
| (Intercept) | 3.2674293 | 3.2008758 | 1.021 | 0.32166 |
| RAI_Pre | -0.0065751 | 0.0089521 | -0.734 | 0.47267 |
| RAI_small | -0.0001189 | 0.0002124 | -0.560 | 0.58295 |
| RAI_large | 0.0089892 | 0.0078586 | 1.144 | 0.26853 |
| H_Pre | 0.9567745 | 2.6996058 | 0.354 | 0.72739 |
| H_small | -1.4062593 | 2.2150009 | -0.635 | 0.53396 |
| H_large | 5.9295704 | 1.9836412 | 2.989 | 0.00824** |
| S_all | -0.2959059 | 0.3693558 | -0.801 | 0.43411 |

Significance: 0 ‘***’ 0.001 ‘**’ 0.01 ‘*’ 0.05 ‘.’ 0.1 ‘ ’ 1

**Table S7.** Conditional averaged linear regression results using a normal distribution for effects of Shannon diversity of larger non-predatory mammals and predators and total species richness of mammals on *Borrelia* genospecies diversity. Significant variables are marked with an*.

| Predictor | Estimate | Std. Error | p-value |
| --- | --- | --- | --- |
| (Intercept) | 1.6341 | 2.7174 | 0.55829 |
| H_large | 5.8693 | 1.7806 | 0.00171* |
| S_all | -0.3899 | 0.2712 | 0.17434 |
| H_Pre | 2.1762 | 2.0123 | 0.30677 |

**Table S8.** AICc table for the candidate models describing *Borrelia* genospecies diversity, displaying the models with delta AICc<2 (Full model: *Borrelia* genospecies diversity ~ RAI_Pre + H_Pre + RAI_large + H_large + RAI_small + H_small + S_all)

| Candidate models - Borrelia *genospecies diversity* | AICc | Delta AICc | Weight |
| --- | --- | --- | --- |
| Intercept + H_large | 111.1 | 0.00 | 0.146 |
| Intercept + H_large + S_all | 111.7 | 0.61 | 0.107 |
| Intercept + H_large + H_Pre | 112.7 | 1.56 | 0.067 |

**Table S9.** Results of the full linear regression model for effects of the relative abundance indices and Shannon diversities of predators, small and large mammals as well as the total species richness of mammals on *Borrelia* sequence type diversity (log(+1)-transformed).

|  | Estimate | Std. Error | t value | Probability (>\|t\|) |
| --- | --- | --- | --- | --- |
| (Intercept) | 1.019e+00 | 1.054e+00 | 0.967 | 0.347 |
| RAI_Pre | -5.865e-04 | 2.947e-03 | -0.199 | 0.845 |
| RAI_Sum_Apo | -3.106e-05 | 6.992e-05 | -0.444 | 0.663 |
| RAI_Mult_large | 5.361e-04 | 2.587e-03 | 0.207 | 0.838 |
| H_Pre | -3.907e-01 | 8.886e-01 | -0.440 | 0.666 |
| H_small | -1.732e-01 | 7.291e-01 | -0.238 | 0.815 |
| H_Mult_large | 6.123e-01 | 6.529e-01 | 0.938 | 0.361 |
| S_all | -7.345e-03 | 1.216e-01 | -0.060 | 0.953 |

**Table S10.** Conditional averaged linear regression results using a normal distribution for effects of Shannon diversity of larger non-predatory mammals on *Borrelia* sequence type diversity (log(+1)-transformed).

| Predictor | Estimate | Std. Error | p-value |
| --- | --- | --- | --- |
| (Intercept) | 0.7852 | 0.2134 | 0.00041*** |
| H_large | 0.4277 | 0.4836 | 0.40205 |

**Table S11.** AICc table for the candidate models describing *Borrelia* sequence type (ST) diversity, displaying the models with delta AICc<2 (Full model: *Borrelia* ST diversity ~ RAI_Pre + H_Pre + RAI_large + H_large + RAI_small + H_small + S_all)

| **Candidate models - *Borrelia* ST** ***diversity*** | **AICc** | **Delta AICc** | **Weight** |
| --- | --- | --- | --- |
| Intercept | 48.5 | 0.00 | 0.182 |
| Intercept + H_large | 50.2 | 1.76 | 0.076 |
